# Prediction of new drug indications based on clinical data and network modularity

**DOI:** 10.1038/srep32530

**Published:** 2016-09-28

**Authors:** Liang Yu, Xiaoke Ma, Long Zhang, Jing Zhang, Lin Gao

**Affiliations:** 1 School of Computer Science and Technology, Xidian University, Xi’an, 710071, P. R. China; 2 Department of Sports, Xidian University, Xi’an, 710071, P. R. China

## Abstract

Drug repositioning is commonly done within the drug discovery process in order to adjust or expand the application line of an active molecule. Previous computational methods in this domain mainly focused on shared genes or correlations between genes to construct new drug-disease associations. We propose a method that can not only handle drugs or diseases with or without related genes but consider the network modularity. Our method firstly constructs a drug network and a disease network based on side effects and symptoms respectively. Because similar drugs imply similar diseases, we then cluster the two networks to identify drug and disease modules, and connect all possible drug-disease module pairs. Further, based on known drug-disease associations in CTD and using local connectivity of modules, we predict potential drug-disease associations. Our predictions are validated by testing their overlaps with drug indications reported in published literatures and CTD, and KEGG enrichment analysis are also made on their related genes. The experimental results demonstrate that our approach can complement the current computational approaches and its predictions can provide new clues for the candidate discovery of drug repositioning.

Traditionally, drug discovery process mainly consists of three stages: discovery, preclinical stage, and clinical development^1^. In the discovery stage, new drugs are screened and identified. Then, the new drugs are tested *in vitro* and in animal models in the preclinical stage. Finally, the drug candidates are tested in human beings as clinical trials in the clinical development stage. The whole process is time-consuming, costly, and often inefficient. It is conservatively estimated that the time for bring a drug to market is about 15 years^2^, and the cost is $800~1000 million^3^. Drug repositioning, which refers to identify and use the known drugs that can treat diseases other than those for which they were originally designed^4^, is an increasingly attractive mode of therapeutic discovery. This strategy certainly has the potential of being the most efficient technique for drug development since it does not need the initial six to nine years, thus reducing risk and costs^5^. There are a number of drug examples that have been successfully repositioned such as sildenafil citrate (brand name: Viagra), which was repositioned from a common angina drug to a therapy for erectile dysfunction and pulmonary hypertension^6^, and minoxidil which was originally tested for hypertension and now was indicated for hair loss^7^.

With the dramatic expansion of available high-throughput datasets, many approaches to discover new drug indications have been developed. Some are mainly focused on small-scale applications to analyze specific classes of drugs or drugs for specific diseases^8^,^9^,^10^. For example, based on chemical similarity, Noeske *et al*. considered the pharmacophore descriptors of drugs to cluster drugs^8^. In 2010, Kotelnikova *et al*. firstly constructed signaling pathways related to glioblastoma based on data got from scientific literature and ResNet database. Then using Sub-Network Enrichment Analysis (SNEA), they analyzed the differential expression in glioblastoma patients^9^. There are also a few examples involving a relatively large number of drugs and diseases^11^,^12^,^13^,^14^,^15^. Lamb *et al*.^11^ created the first installment of a reference collection of gene-expression profiles to discovery functional connections among diseases, genetic perturbation, and drug action. Based on molecular activity similarity, the researchers constructed a drug network^12^ and they partitioned the network into densely interconnected groups. The drugs in the same group are significantly enriched for compounds with similar mode of action, or in the same pathway, which can be used to identify the compound-targeted biological pathways. Some methods also predicted drug-target interactions for drug repositioning^16^,^17^ and microRNA-disease associations based on social network analysis methods^18^,^19^. In 2014, Ye *et al*. constructed drug-drug interaction through side effect similarities and predicted the indications of a drug by the functions of its neighboring drugs^20^. PREDICT is based on the observation that similar drugs are indicated for similar diseases, and utilizes multiple drug-drug and disease-disease similarity measures for the prediction of drug-disease associations^21^. It allows easy integration of additional similarity measures among diseases and drugs.

Here, we propose a method for predict potential drug-disease associations that can not only handle drugs or diseases with or without related genes but consider the network modularity. The main framework of our method is shown in Fig. 1. Based on side effects of drugs and symptoms of diseases, drug-drug and disease-disease weighted networks are firstly constructed. Then, we cluster the two networks and get drug-module and disease-module sets. Further, according to the known associations in Comparative Toxicogenomics Database (CTD; http://ctd.mdibl.org)^22^ between drugs and diseases, we correlate drug and disease modules with scores. Finally, we rank the drug-disease module pairs according to their scores and select the top-3 drug-disease module pairs for further analysis. For each selected drug-disease module pair, we score the connection between each drug and each disease in the pair. The larger the score, the greater the degree of reliability, thus the greater the possibility of drug relation to disease. We evaluate our predictions with drug-disease associations that are reported in published literature and CTD benchmark, and also make Kyoto Encyclopedia of Genes and Genomes (KEGG) enrichment analysis on their related genes. The results demonstrate that our predictions can provide new clues for drug repositioning based on drug side effects, disease symptoms and network modularity.

## Results

Based on the top-3 drug-disease module pairs, shown in Table 1, we get three ranking lists of drug-disease association and predict new drug indications. The top associations in each list are reliable. Therefore, we evaluate the top-20 predicted associations in each list by their overlap with drug indications that are reported in published literature and Comparative Toxicogenomics Database (CTD; http://ctd.mdibl.org) benchmark, and we also make Kyoto Encyclopedia of Genes and Genomes (KEGG) enrichment analysis on them.

### CTD benchmark and literature verification

Table 2, 3, 4 respectively show three lists of top-20 drug-disease associations corresponding to the three drug-disease module pairs in Table 1 (Rank = 1 to 3). In these tables, the predicted results are divided into two categories: known and potential associations. If there is direct evidence for a chemical-disease association marked as “therapeutic” or “marker/mechanism” in CTD database, we take it as a known association. Otherwise, it is considered as a potential drug-disease association (marked as bold italic items), such as prochlorperazine and Tremor (ID = 9) in Table 2. In the following section, we abbreviate “therapeutic” to “T”, and “marker/mechanism” to “M”.

#### Verification of top-20 drug-disease associations in module pair while Rank = 1

**Figure 2:**
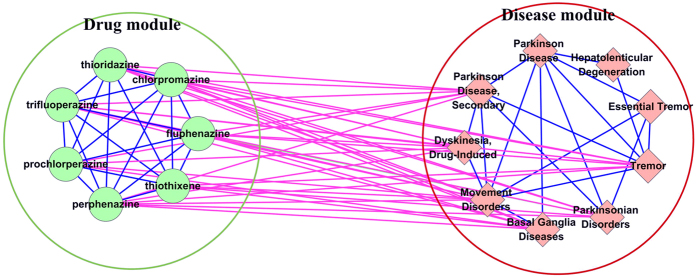
The network topology of the first drug-disease module pair (Rank = 1 in Table 1). Internal connections within a module are labelled by blue, and external connections between two modules are labelled by purple. Green circle and red diamond nodes represent drugs and diseases respectively. The purple edges represent the reliably curated drug-disease associations in CTD (CTD mark is “M” or “M&T”).

Figure 2 shows the network topology of the first drug-disease module pair (Rank = 1 in Table 1). In the figure, internal connections within a module are labelled by blue, and external connections between two modules are labelled by purple. Green circle and red diamond nodes represent drugs and diseases, respectively. The purple edges represent the reliably curated drug-disease associations in CTD, which are also shown in Table 2 (CTD mark are “M” or “M&T”).

In Table 2, we find 18 of 20 are known associations. The percentage reaches up to 90%. Two results are new predictions and labelled by bold italic. The association between fluphenazine and Parkinsonian Disorders (ID = 19) is inferred by CTD (Inference Score = 7.38). The inference score^23^ reflects the degree of similarity between CTD chemical-gene-disease networks and a similar scale-free random network, which is computed as shown below:


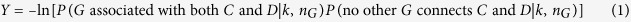


where *Y* represents the inference score; *P* represents the probability that a vertex in a large network interacts with another vertex decays according to a power law^24^; *G*, *C*, and *D* represent a gene, chemical, and disease respectively; *k* represents the number of connection between *G*, *C*, or *D*; *n*_*G*_ represents a gene set. The higher the *Y* score, the more likely the inference network has atypical connectivity^25^.

We also find the inferred association (ID = 19) is based on three genes: NGF^26^, DRD2^27^, and PRL^28^. Moreover, the two results (in bold italic) are also supported by external literature. Studies^29^ have shown that prochlorperazine can cause Tremor (ID = 9). People possibly have tremor while taking prochlorperazine from FDA (Food and Drug Administration) and social media. Fluphenazine (ID = 19) is a drug used to treat psychotic disorders, agitation, and dementia^30^. And its use may lead to the development of symptoms that resemble Parkinson’s disease^31^.

#### Verification of top-20 drug-disease associations in module pair while Rank = 2

Supplementary Fig. S1 shows the relationships of the second drug-disease module pair (Rank = 2 in Table 1). Table 3 gives the details of top-20 drug and disease associations. In this table, sixteen associations are known and the remaining four ones marked as bold italic are new predicted results. The association between thorazine and Lewy Body Disease (ID = 16) is inferred associations in CTD (Inference Score = 3.77). The relationship is based on the gene “MAG”. Evidences show that thorazine affects the expression of MAG mRNA^32^ and that MAG expression significantly relates to Lewy Body Disease^33^. Thorazine is an antipsychotic medication, which is primarily used to treat psychotic disorders such as schizophrenia. Lewy body dementia (LBD) is a type of progressive dementia that leads to a decline in thinking, reasoning and independent function because of abnormal microscopic deposits that damage brain cells over time^34^. LBD is found among people who take thorazine, especially for people who are male, more than 60 years old, also take medication protonix, and have insomnia^35^. The researchers found thorazine had shown better capacity to control hyperkinetie manifestations, and had not shown any undesirable side effects^36^,^37^.

For the association (ID = 10), thioridazine is a typical antipsychotic drug used in the treatment of Psychoses (ID = 10)^38^. Prochlorperazine (ID = 18) belongs to a group of medicines called “phenothiazines”. It is prescribed for a variety of unrelated conditions, including problems with balance and dizziness, sickness, agitation and restlessness, and schizophrenia^39^. Bipolar Disorder (ID = 18) is found among people who take prochlorperazine, especially for people who are female, more than 60 years old, also take medication klonopin, and have nausea^40^. Symptoms typical of alcohol withdrawal (ID = 20) include agitation, seizures, and delirium tremens^41^. Laties and colleagues^42^ determined that promazine and chlorpromazine (ID = 20) were equally efficacious in the treatment of delirium tremens. Chlorpromazine, promazine, and other low-potency typical antipsychotic agents have been reported^43^ to have the greatest effect on lowering seizure threshold.

#### Verification of top-20 drug-disease associations in module pair while Rank = 3

The network topology of the third drug-disease module pair (Rank = 3 in Table 1) is shown in Supplementary Fig. S2. From Table 4, we find 2 associations (in bold italic) are new predictions by our method. The other 18 associations are all known in CTD. The percentage reaches up to 90%. Though, two other associations (ID = 15 and ID = 20) are not existed in CTD at present, their associations are supported by features of their ancestors and literatures. Circadian rhythms (“body clocks”) are controlled by a biological clock and work on a daily time scale. Circadian rhythm sleep disorders (CRSD) (ID = 15) are a family of sleep disorders. People with circadian rhythm sleep disorders are unable to sleep and wake at the times required for normal work, school, and social needs. We find amitriptyline (ID = 15) has strong connections with sleep disorders in CTD (marked as “M&T”). They are related by the gene “CHRNB2”: amitriptyline results in the decreased expression of CHRNB2 mRNA^44^ and CHRNB2 protein results in the increased susceptibility to amitriptyline^45^. Hence, it is reasonable to infer that circadian rhythm sleep disorders and amitriptyline probably have close connection. Moreover, amitriptyline is found useful as a sleep aid continues^46^. Postpartum depression (ID = 20) is moderate to severe depression in a woman after she has given birth, which is a descendant of depressive disorder. The relationship between imipramine (ID = 20) and depressive disorder is marked as “therapeutic” and “marker/mechanism”, which is based on five genes: ALB^47^, BDNF^48^, CRH^49^, POMC^50^, SLC6A4^51^. Therefore, it is likely that postpartum depression has connection with imipramine. Furthermore, Cohen and Rosenbaum^52^ pointed out the use of tricyclic antidepressants should not pose a risk when used in pregnancy or in the postpartum period. They maintained that the safest medications to use at this point in time are nortriptyline, imipramine, and fluoxetine.

### KEGG pathway enrichment analysis

In this section, for the predicted drug-disease associations shown in bold italic in above tables, we perform KEGG pathway enrichment analysis on their related gene sets with the functional annotation tool of DAVID^53^. For DAVID, EASE Score, a modified Fisher Exact P-Value, is used as a threshold for gene-enrichment analysis^54^. It ranges from 0 to 1. When Fisher Exact P-Value is 0, it represents perfect enrichment. We set it as 0.01.

For each drug, we combine the genes obtained from Drugbank database^55^, which combines detailed drug data with comprehensive drug target information, and the top interacting genes got from CTD database. Similarly, the genes related to each disease are got from OMIM database^56^, which is a comprehensive, authoritative compendium of human genes and genetic phenotypes.

For each predicted drug and disease pair, we respectively put their corresponding genes into DAVID and examine whether the drug and the disease have overlapped KEGG pathways. If they have more overlapped pathways, they are more relevant, that is the drug has strong correlation with the disease. For example, prochlorperazine and tremor (Rank = 9 in Table 2) has one overlapped pathway: “Neuroactive ligand-receptor interaction” (p-value = 1.2E-3). We find thioridazine (Rank = 10 in Table 3) has two pathways overlapping with psychoses, substance-induced: “neuroactive ligand-receptor interaction” and “calcium signaling pathway”. Their corresponding p-values are 2.14E-05 and 2.81E-04 respectively. Lower p-values indicate the predicted associations are reliable. Especially, the pathway overlapped between amitriptyline and dyssomnias (Rank = 16 in Table 4) is “neuroactive ligand-receptor interaction”, which has a very low p-value: 8.66E-29. These good results show their high reliability. Because of the incompleteness of data, the numbers of genes related to some drugs and diseases are all small, such as prochlorperazine and bipolar disorder (Rank = 18) in Table 3. Therefore, it is hard to find pathways related to prochlorperazine and bipolar disorder using DAVID tool at present. With the improvement of data, the performance of our method will be more effectively.

### Comparison with other method based on CTD benchmark

To evaluate the performance of our method, we compare it with PREDICT^21^. PREDICT integrates multiple data sources, including chemical structures, drug side effects, drug target protein sequences and target protein interactions and phenotype data. It applies network analysis for target protein distance calculation, applies text mining to identify disease phenotypes and use machine learning algorithms to classify true and false drug-disease associations^2^.

Based on CTD benchmark, we make a comparison between our method and PREDICT^21^. We choose the top-20 drug-disease associations of PREDICT for analysis. They are shown in Table 5. In the table, the column “Disease name” sometimes includes more than one disease name. As long as one disease is found related to a drug in CTD, the association is marked as “T”, “M”, “M&T” or “inferred”. Taking the fourth association (Rank = 4) as an example, its “Disease name” = “Pyogenic Sterile Arthritis, Pyoderma Gangrenosum, And Acne” (Rank = 4). We find “Arthritis” is related to “Gonadorelin” in CTD and it is an inferred association. Thus the association is marked as “inferred”, i.e. CTD mark = “inferred”. Finally, there are 14 drug-disease connections are found in CTD benchmark, but only 5 of them are known associations (CTD mark = “T” or “M&T” and marked as bold italic in Table 5) and the other 9 associations are inferred in CTD (CTD mark = “inferred”). Its precision is 5/20 = 0.25, which is lower than that of our method. The precision of our top-20 associations in each list is more than 0.8. From Table 2, 3, 4, they are 0.9, 0.8, and 0.9 respectively.

## Discussion

Based on side effects of drugs and symptoms of diseases, we construct drug and disease networks firstly. Then we cluster them to get two types of modules: drug module and disease module. According to the known drug-disease associations in CTD database, we score each pair of drug-disease module and reserve the top-3 drug-disease module pairs. For each selected drug-disease module pairs, we construct a drug-disease bipartite graph. We calculate all the connections to predict potential drug-disease associations. The significant enrichments of our predictions in the biomedical literature, clinical trials and KEGG pathways demonstrate that our approach can effectively identify new indications as an indicator for the mode of action. The success of our methods PDCIM can be attributed as follows: First, we integrate clinical data into our model, such as disease symptoms and drug side effects. Second, our approach is based on clustering and known drug-disease associations. Finally, we combine the neighborhood information of nodes in drug modules and disease modules. We believe that the combination of clinical data, network clustering and subnetwork connectivity could help us to predict new hypotheses to infer the drug-disease relationship and even improve the drug development. Moreover, our predicted drug-disease associations are not generated using the genes associated with diseases or drugs, so we can find some drug-disease associations that these drugs or diseases have less or no related genes. However, the limitation is the difficulty in distinguishing the positive and negative associations between drugs and diseases. In the future, we can choose different methods to calculate the similarity between drugs and diseases to enhance the reliability of our constructed networks. On the other hand, we can integrate various data sources such as pharmacological data, therapeutic/toxicological expression profiles and DNA methylation data to try to distinguish the positive and negative associations between drug and disease.

## Methods

### Data Source

#### Human symptom-disease data

The human symptom-disease data is got from^57^. Based on 322 disease symptoms, a weighted disease network is constructed, which contains 133,106 interactions between 1,596 distinct diseases (see Supplementary Table S1). The disease network combines phenotypic relations with shared molecular mechanisms.

#### Drug-side effect data

The drug-side effect data is downloaded from SIDER (Side Effect Resource) version 2^58^. SIDER contains information on 996 marketed drugs, corresponding 4,192 recorded adverse drug reactions, and 99,423 drug-side effect pairs (see Supplementary Table S2). The information is extracted from public documents and package inserts. The available information include side effect frequency, drug and side effect classifications as well as links to further information, for example drug–target relations.

#### Drug-Disease data

We obtain the drug–disease associations from Comparative Toxicogenomics Database (CTD) in December 2015^22^. CTD contains curated and inferred chemical–disease associations. They can help researchers develop hypotheses about environmental diseases and their underlying mechanisms. We only preserve the curated associations with marker “therapeutic” or “marker/mechanism” as known associations. “Therapeutic” represents that a chemical has a known or potential therapeutic role in a disease (e.g., chemical X is used to treat leukemia). “Marker/mechanism” represents that a chemical correlates with a disease (e.g., increased abundance in the brain of chemical X correlates with Alzheimer disease) or may play a role in the etiology of a disease (e.g., exposure to chemical X causes lung cancer)^22^. Finally, we get 82,858 chemical–disease associations in all (see Supplementary Table S3).

### Construct drug similarity network based on side effects

The relationship between drug *j* and side effects is treated as a feature vector *d*_*j*_:


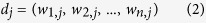


where *w*_*i,j*_ quantifies the strength of the association between side effect *i* and drug *j*. The prevalence of the different side effects and diseases is very different. For example, there are highly abundant side effects like abdominal pain, and publication biases towards certain drugs. To account for this heterogeneity, we do not use the absolute co-occurrence *W*_*i,j*_ to measure the strength of an association between side effect *i* and drug *j*, but the term frequency-inverse document frequency^59^ *w*_*i,j*_:


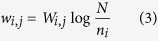


where *N* denotes the number of all drugs in the dataset and *n*_*i*_ denotes the number of drugs in which side effect *i* appears. *W*_*i,j*_ is equal to 1, if drug *j* displays side effect *i*, otherwise, it will be 0. Since all side effects in our data have at least one associated drug, the potential problem of dividing by zero does not arise. For the 996 drugs with recorded clinical side effect data, each will be assigned a 4192-dimension vector.

We use the cosine similarity^60^ to measure the similarity between the vectors *d*_*i*_ and *d*_*j*_ of two drugs *i* and *j*. The formula is shown as:


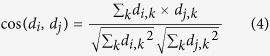


where the cosine similarity ranges from 0 (no shared side effects) to 1 (identical side effects).

### Cluster constructed networks using ClusterONE

After filtering drug and disease networks, we respectively cluster drug network and disease network using ClusterONE (Clustering with Overlapping Neighborhood Expansion)^61^ and obtain two kinds of modules: drug module and disease module. ClusterONE is a graph clustering algorithm that is able to handle weighted graphs. Owing to these properties, ClusterONE is especially useful for detecting modules in networks with associated confidence values.

#### Cluster drug similarity network

**Figure 3:**
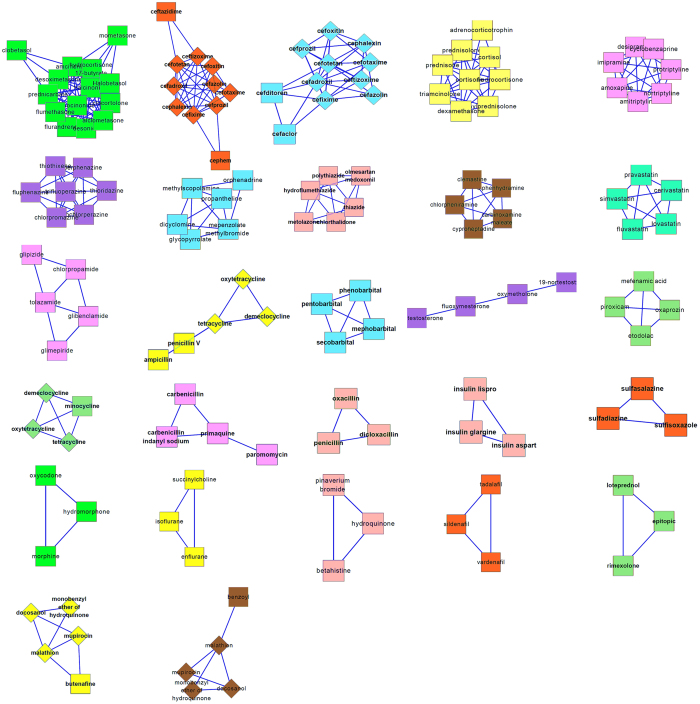
The network topologies of 27 modules, here nodes representing drugs; rectangles represent nodes in clusters; and diamonds represent overlap nodes between different clusters.

In order to improve the reliability of the drug network, we filter the edges with lower similarity. To remain relatively more reliable edges, the cutoff is set to be 0.4 and we get a new drug network with 248 nodes and 379 edges, as shown in Supplementary Table S4. We run ClusterONE with default parameter values in the filtered drug network and get 27 modules (See Supplementary Table S5). The p-values of 26 modules are lower than 0.05. The network topologies of 27 modules are also shown in Fig. 3 (nodes representing drugs). Rectangles and diamonds represent nodes in clusters and overlap between different clusters, respectively.

#### Cluster disease similarity network

When the scores of edges in disease network are not lower than 0.5, we can obtain more meaningful modules (p–value ≤ 0.05). Therefore, we discard the edges with scores lower than 0.5. After filtering, the disease network includes 1,367 nodes and 9,792 edges, as shown in Supplementary Table S6. We also run ClusterONE with default parameter values in disease network and get 145 modules (see Supplementary Table S7). The p-values of 64 modules are lower than 0.05.

### Construct connections between disease and drug modules based on CTD

**Figure 4:**
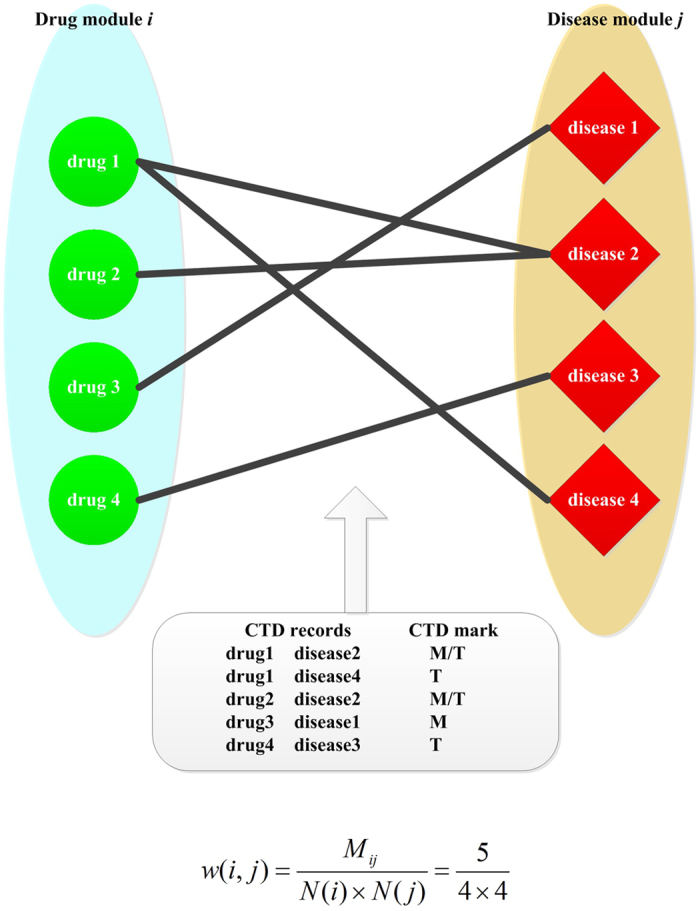
An example of calculating the correlation between drug module *i* and disease module *j* based on the drug-disease associations verified by CTD (marked as “M” or “M&T”).

Based on reliable chemical-disease associations got from CTD database, we construct connections between disease and drug modules. As an example shown in Fig. 4, we assume drug module *i* includes four drugs (drug1 to drug4) and disease module *j* includes four diseases (disease1 to disease4). The strength of the association between drug module *i* and disease module *j*, *w*(*i*, *j*), is defined as:


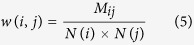


where *M*_*ij*_ denotes the number of chemical-disease associations verified by CTD (marked as T or M/T) between drug module *i* and drug module *j*; *N*(*i*) and *N*(*j*) denote the number of elements in drug module *i* and drug module *j*, respectively. In Fig. 4, *M*_*ij*_ = 5 and *N*(*i*) = *N*(*j*) = 4, so 
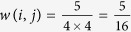
. In this way, we can get the correlation between any drug-disease module pair.

Finally, we obtain 1,180 drug-disease module pairs whose score are not zero. According to the definition of the association between a drug-disease module pair (see formula (5)), the drug-disease module pairs with higher score will be preserved. There are 27 drug-disease module pairs whose scores are all not lower than 0.2, which are shown in Supplementary Table S8. In order to analyze our results more targeted and find more valuable associations, we focused on the top-3 drug-disease module pairs for further analysis. The top-3 drug-disease module pairs whose correlations are all not lower than 0.36. Their details are shown in Table 1. In Results section, we will analyze the top-3 drug-disease module pairs to predict new drug indications.

### Predict drug-disease associations based on top-3 drug-disease module pairs

In this section, we predict novel drug-disease associations based on the neighborhood partnerships of drugs and diseases in their own modules. Table 1 shows the information of top-3 drug-disease module pairs. We can find these modules are dense, that is to say, the nodes in a same module interact frequently. We know that the stronger the interactions between nodes within a same module, the more similar their functions. Therefore, based on the strong correlations between dense drug and disease modules, we can predict potential associations between drugs and diseases. The details are as following:

Step 1: Recalculate the similarity of each pair of nodes in a module based on subgraph topology of the module. Given ***a*** and ***b*** is a pair of nodes in a drug module or a disease module, there may be many different paths between ***a*** and ***b***. Suppose ***path***_***i***_ represents a path between ***a*** and ***b*** with ***i*** edges; ***sum***(***path***_***i***_) represents the weight of ***path***_***i***_, which equals to the sum of weight on each edge in it; ***avg***(***path***_***i***_) represents the average weight of ***path***_***i***_: ***avg***(***path***_***i***_) = ***sum***(***path***_***i***_)/***i***. Then, the similarity between ***a*** and ***b*** is defined as:


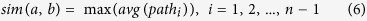


where ***n*** represents the number of nodes in the module. If there does not exist a path with ***i*** edges, we set ***avg***(***path***_***i***_) = 0. In this way, we score all the correlations between each pair of nodes in a module.

From the above steps, we know drugs or diseases have similar properties are clustered in a same group. The indications of a drug may be inferred by the enriched FDA-approved functions of its neighboring drugs in a same drug subgraph. Relationships in strong similar edges and connected components provide potential candidates for the previously unknown therapeutic effects of drugs. In the same way, the treatments of a disease may be inferred by its neighboring diseases in a same disease subgraph. Therefore, for a drug-disease module pair, we predict new drug-disease associations based on the subgraph topology of modules.

Step 2: Recalculate the association of each pair of nodes belong to two different modules based on Step 1. Based on known drug-disease associations in CTD database, and correlations of drug-drug and disease-disease obtained from Step 1, we get a drug-disease heterogeneous network, which includes two types of nodes: drug and disease, and two types of edges: internal connections and external connections. Then, based on the heterogeneous network, we score the correlations between each pair of drug and disease nodes. Given ***v*** represents a node in disease module ***DI*** and ***w*** represents a node in drug module ***DR***, that is ***v***∈***DI*** and ***w***∈***DR***. Their relation **c*****orr***(***v***, ***w***) is defined as:


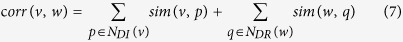


The direct neighbors of ***v*** in ***DI***, which have connections with nodes in ***DR***, are stored in ***N***_***DI***_(***v***). The direct neighbors of ***w*** in ***DR***, which have connections with nodes in ***DI***, are stored in ***N***_***DR***_(***w***). ***sim***(***v***, ***p***) and ***sim***(***w***, ***q***), defined as Equation (6), denote the similarities between ***v*** and ***p***, ***w*** and ***q***, respectively.

After the process of Step 1 and Step 2, we will score all the drug-disease associations between a drug-disease module pair and rank them in descending order. We do the same process for the top-3 drug-disease module pairs and get three ranking lists of drug-disease associations.

## Additional Information

**How to cite this article**: Yu, L. *et al*. Prediction of new drug indications based on clinical data and network modularity. *Sci. Rep.* **6**, 32530; doi: 10.1038/srep32530 (2016).

## Supplementary Material

Supplementary Information

Supplementary Table S1

Supplementary Table S2

Supplementary Table S3

Supplementary Table S4

Supplementary Table S5

Supplementary Table S6

Supplementary Table S7

Supplementary Table S8

## Figures and Tables

**Figure 1:**
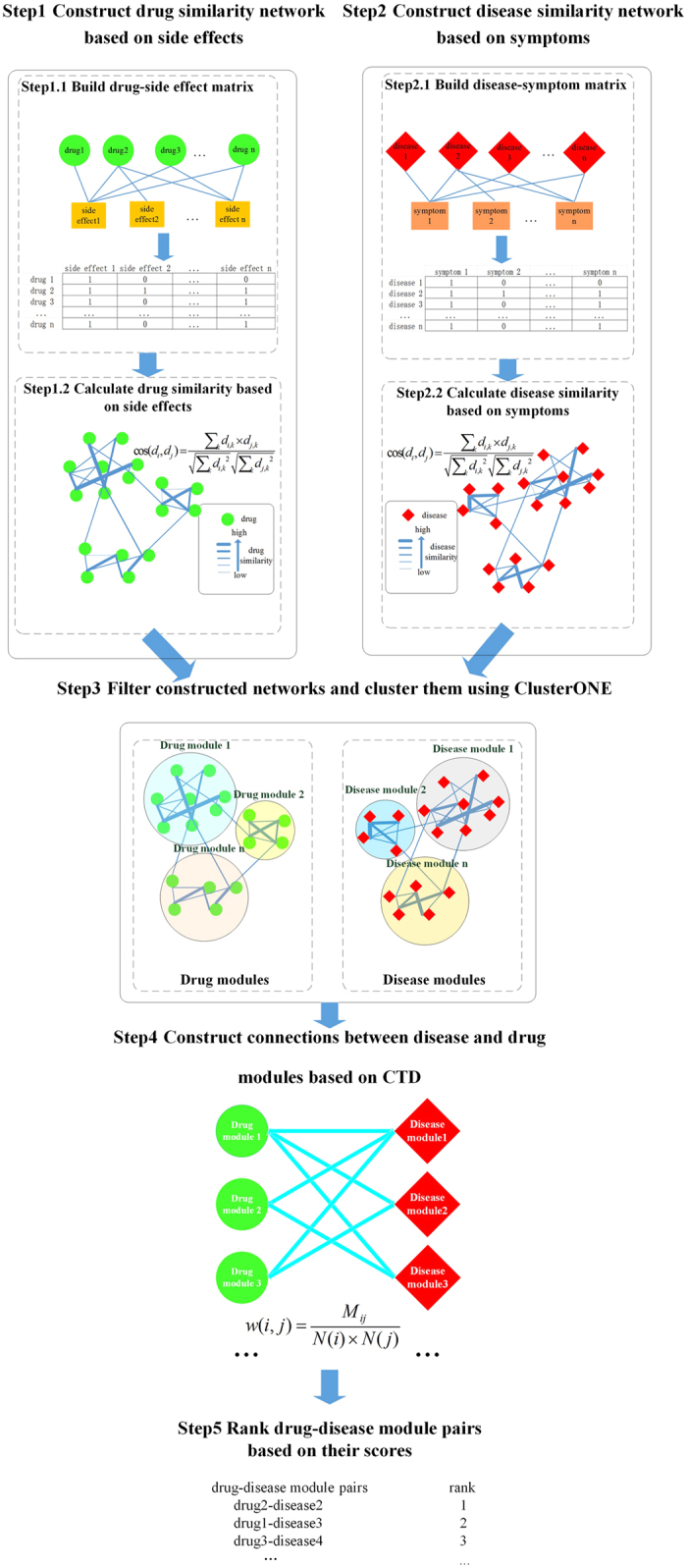
The framework of our method.

**Table 1:** Information of top-3 drug-disease module pairs.

Rank	Drugs in drug module	Diseases in disease module	Number of drugs	Number of diseases	Density of drug module	Density of disease module	Score between drug-disease module pair
1	thiothixene,	Parkinson Disease Secondary,					
		Tremor,					
	chlorpromazine,	Dyskinesia Drug-Induced,					
	fluphenazine,	Movement Disorders,					
	perphenazine,	Parkinsonian Disorders,	7	9	0.95	0.50	0.59
	prochlorperazine	Basal Ganglia Diseases,					
	thioridazine,	Essential Tremor,					
	trifluoperazine	Parkinson Disease,					
		Hepatolenticular Degeneration					
2	thiothixene,	Alcohol Withdrawal Delirium,					
	chlorpromazine,	Schizophrenia,					
	fluphenazine,	Substance-Related Disorders,					
	perphenazine,	Bipolar Disorder,					
	prochlorperazine	Narcolepsy,	7	8	0.95	0.71	0.39
	thioridazine,	Lewy Body Disease,					
	trifluoperazine	Psychoses Substance-Induced,					
		Psychotic Disorders					
3		Alcoholism,					
		Depressive Disorder Major,					
	protriptyline,	Mood Disorders,					
	imipramine,	Depressive Disorder,					
	cyclobenzaprine,	Schizophrenia,					
	nortriptyline,	Substance-Related Disorders,					
	amoxapine,	Bipolar Disorder,	7	12	0.95	0.50	0.36
	desipramine,	Personality Disorders,					
	amitriptyline	Anxiety Disorders,					
		Depression Postpartum,					
		Dyssomnias,					
		Sleep Disorders Circadian Rhythm					

**Table 2:** Top-20 drug and disease associations in drug-disease module pair while Rank = 1.

Rank	Drug Name	Disease Name	Score	CTD mark
1	trifluoperazine	Basal Ganglia Diseases	7.36	M
2	thorazine	Basal Ganglia Diseases	7.15	M&T
3	perphenazine	Basal Ganglia Diseases	7.01	M
4	trifluoperazine	Movement Disorders	6.92	M
5	trifluoperazine	Dyskinesia, Drug-Induced	6.81	M
6	trifluoperazine	Parkinson Disease, Secondary	6.73	M
7	Thorazine	Movement Disorders	6.71	M&T
8	Thorazine	Dyskinesia, Drug-Induced	6.62	M&T
***9***	***prochlorperazine***	***Tremor***	***6.59***	***none***
10	perphenazine	Movement Disorders	6.56	M&T
11	Thorazine	Parkinson Disease, Secondary	6.54	M
12	thioridazine	Basal Ganglia Diseases	6.44	M
13	perphenazine	Dyskinesia, Drug-Induced	6.43	M
14	perphenazine	Parkinson Disease, Secondary	6.36	M
15	prochlorperazine	Parkinsonian Disorders	6.34	M
16	perphenazine	Tremor	6.22	M
17	trifluoperazine	Tremor	6.22	M
18	Thorazine	Tremor	6.18	M
***19***	***fluphenazine***	***Parkinsonian Disorders***	***6.06***	***inferred***
20	prochlorperazine	Basal Ganglia Diseases	6.03	M

CTD mark represents a drug-disease association is curated, inferred or not existed in CTD database. Curated associations include three types: marker/mechanism (CTD mark = “M”), therapeutic (CTD mark = “T”), marker/mechanism & therapeutic (CTD mark = “M&T”). If an association is inferred by CTD, CTD mark = “inferred”, and if it is not existed in CTD, CTD mark = “none”.

**Table 3:** Top-20 drug and disease associations in drug-disease module pair while Rank = 2.

Rank	Drug name	Disease name	Score	CTD mark
1	chlorpromazine	Psychotic Disorders	5.36	T
2	chlorpromazine	Schizophrenia	5.19	T
3	chlorpromazine	Psychoses, Substance-Induced	5.19	M&T
4	perphenazine	Schizophrenia	5.15	T
5	Thorazine	Bipolar Disorder	5.07	M&T
6	thiothixene	Psychotic Disorders	4.91	M&T
7	trifluoperazine	Psychotic Disorders	4.72	T
8	trifluoperazine	Psychoses, Substance-Induced	4.58	M
9	trifluoperazine	Schizophrenia	4.57	T
***10***	***thioridazine***	***Psychoses, Substance-Induced***	***4.56***	***none***
11	trifluoperazine	Bipolar Disorder	4.54	T
12	fluphenazine	Psychotic Disorders	4.42	T
13	fluphenazine	Schizophrenia	4.24	T
14	fluphenazine	Bipolar Disorder	4.16	T
15	fluphenazine	Psychoses, Substance-Induced	4.10	M
***16***	***thorazine***	***Lewy Body Disease***	***4.07***	***inferred***
17	perphenazine	Bipolar Disorder	3.89	T
***18***	***prochlorperazine***	***Bipolar Disorder***	***3.81***	***none***
19	perphenazine	Psychotic Disorders	3.78	T
***20***	***chlorpromazine***	***Alcohol Withdrawal Delirium***	***3.68***	***none***

CTD mark represents a drug-disease association is curated, inferred or not existed in CTD database. Curated associations include three types: marker/mechanism (CTD mark = “M”), therapeutic (CTD mark = “T”), marker/mechanism & therapeutic (CTD mark = “M&T”). If an association is inferred by CTD, CTD mark = “inferred”, and if it is not existed in CTD, CTD mark = “none”.

**Table 4:** Top-20 drug and disease associations in drug-disease module pair while Rank = 3.

Rank	Drug name	Disease name	Score	CTD mark
1	imipramine	Bipolar Disorder	8.80	M&T
2	amitriptyline	Bipolar Disorder	8.51	T
3	imipramine	Depressive Disorder, Major	7.96	M&T
4	amitriptyline	Depressive Disorder, Major	7.86	M&T
5	desipramine	Bipolar Disorder	7.38	M&T
6	amitriptyline	Depressive Disorder	6.93	M&T
7	imipramine	Depressive Disorder	6.67	T
8	amitriptyline	Mood Disorders	6.64	M&T
9	desipramine	Depressive Disorder, Major	6.62	T
10	nortriptyline	Bipolar Disorder	6.59	M
11	imipramine	Mood Disorders	6.47	T
12	amitriptyline	Personality Disorders	6.21	T
13	nortriptyline	Depressive Disorder, Major	6.21	M&T
14	amitriptyline	Anxiety Disorders	5.88	T
***15***	***amitriptyline***	***Sleep Disorders, Circadian Rhythm***	***5.82***	***none***
16	amitriptyline	Dyssomnias	5.77	M&T
17	desipramine	Depressive Disorder	5.49	M&T
18	desipramine	Mood Disorders	5.37	M
19	amitriptyline	Schizophrenia	5.32	T
***20***	***imipramine***	***Depression, Postpartum***	***5.23***	***none***

CTD mark represents a drug-disease association is curated, inferred or not existed in CTD database. Curated associations include three types: marker/mechanism (CTD mark = “M”), therapeutic (CTD mark = “T”), marker/mechanism & therapeutic (CTD mark = “M&T”). If an association is inferred by CTD, CTD mark = “inferred”, and if it is not existed in CTD, CTD mark = “none”.

**Table 5:** Top-20 drug and disease associations of PREDICT.

Rank	Drug name	Disease name	Score	CTD mark
1	Gonadorelin	Endometriosis, Susceptibility To, 1	0.997	inferred
2	Escitalopram	Alcohol Dependence	0.997	inferred
3	Escitalopram	Encephalopathy With Intracranial Calcification, Growth Hormone Deficiency,	0.997	inferred
4	Gonadorelin	Pyogenic Sterile Arthritis, Pyoderma Gangrenosum, And Acne	0.997	inferred
5	Levofloxacin	Helicobacter Pylori Infection, Susceptibility To	0.997	none
6	Levonorgestrel	Acroosteolysis With Osteoporosis And Changes In Skull And Mandible	0.997	none
7	Betamethasone	Asthma, Nasal Polyps, And Aspirin Intolerance	0.997	inferred
8	Gonadorelin	Leiomyoma, Uterine; Ul	0.997	inferred
9	Escitalopram	Encephalopathy, Acute Necrotizing 1, Susceptibility To; Ane1	0.997	none
10	Gonadorelin	Polyps, Multiple And Recurrent Inflammatory Fibroid, Gastrointestinal	0.997	none
***11***	***Gonadorelin***	***Prostate Cancer, Hereditary, 1; Hpc1***	***0.997***	***T***
12	Escitalopram	Spastic Paraplegia, Optic Atrophy, And Dementia	0.997	inferred
13	Escitalopram	Peripheral Neuropathy, Ataxia, Focal Necrotizing Encephalopathy, And Spongy Degeneration Of Brain	0.997	none
14	Ofloxacin	Asthma, Nasal Polyps, And Aspirin Intolerance	0.997	inferred
15	Leuprolide	Hypogonadotropic Hypogonadism	0.997	inferred
***16***	***Betamethasone***	***Growth Retardation, Small And Puffy Hands And Feet, And Eczema***	***0.997***	***T***
***17***	***Medroxyprogesterone***	***Breast Cancer***	***0.997***	***M&T***
18	Betamethasone	Mismatch Repair Cancer Syndrome	0.997	none
***19***	***Prednisolone***	***Asthma, Nasal Polyps, And Aspirin Intolerance***	***0.997***	***T***
***20***	***Escitalopram***	***Panic Disorder 1; Pand1***	***0.997***	***M&T***

CTD mark represents a drug-disease association is curated, inferred or not existed in CTD database. Curated associations include three types: marker/mechanism (CTD mark = “M”), therapeutic (CTD mark = “T”), marker/mechanism & therapeutic (CTD mark = “M&T”). If an association is inferred by CTD, CTD mark = “inferred”, and if it is not existed in CTD, CTD mark = “none”.
