# Author Correction: Prediction of new drug indications based on clinical data and network modularity

**DOI:** 10.1038/s41598-022-07363-5

**Published:** 2022-02-24

**Authors:** Liang Yu, Xiaoke Ma, Long Zhang, Jing Zhang, Lin Gao

**Affiliations:** 1 School of Computer Science and Technology, Xidian University, Xi’an, 710071 P. R. China; 2 Department of Sports, Xidian University, Xi’an, 710071 P. R. China

## Introduction

Correction to: *Scientific Reports* https://doi.org/10.1038/srep32530, published online 28 September 2016

This Article contains an error. To construct drug similarity network based on side effects, the Authors used the data and the method reported by Zhou et al. whose paper is cited as Reference 57 in the Article. Therefore, the wording of the Methods, subsection ‘Construct drug similarity network based on side effects’ closely resembles the method description in Reference 57 designed for symptom-based similarity detection, but this reference was not cited in this section. The Authors wish to acknowledge this prior work and the importance of the approach to their study.

