# Supplementary material for: Prediction of new drug indications based on clinical data and network modularity
Source: Sci Rep. 2016 Sep 28;6:32530. doi: 10.1038/srep32530 (PMC5039412; doi:10.1038/srep32530)
Supplement: Supplementary Information [file srep32530-s1.doc]

**Supplementary Information**

**Prediction of** **new drug indications** **based on clinical data and** **network modularity**

**Liang Yu^1^,*, Xiaoke Ma^1^, Long Zhang^2^, Jing Zhang^1^, Lin Gao^1^**

^1^School of Computer Science and Technology, Xidian University, Xi’an, 710071, P.R.China

^2^Department of Sports, Xidian University, Xi’an, 710071, P.R.China

* [lyu@xidian.edu.cn](mailto:lyu@xidian.edu.cn)


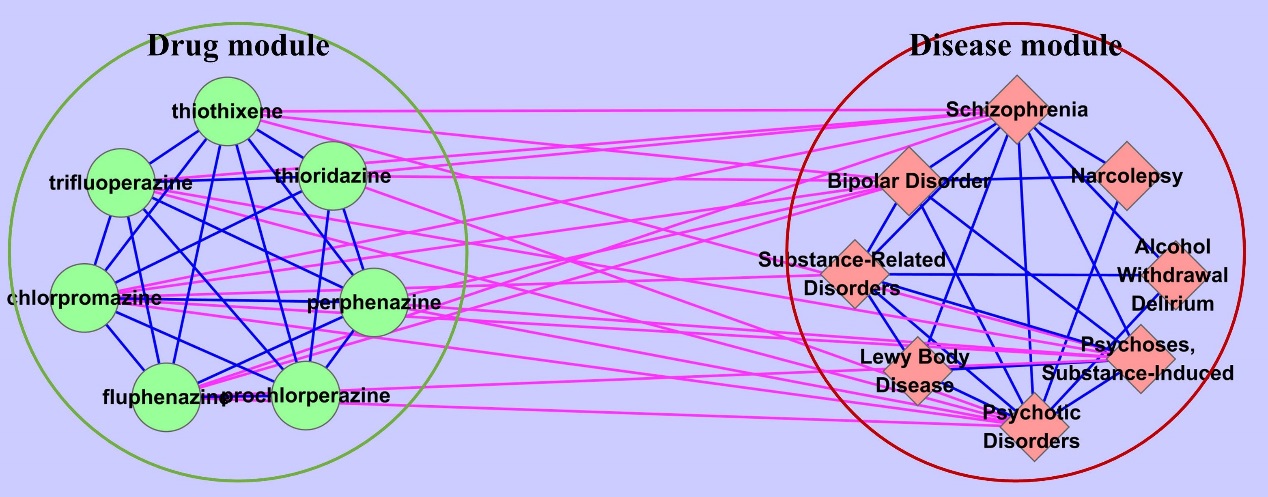


Supplementary Fig S1. The network topology of the second drug-disease module pair in Table 1 (Rank = 2). Internal connections within a module are labelled by blue, and external connections between two modules are labelled by purple. Green circle and red diamond nodes represent drugs and diseases respectively. The purple edges represent the reliably curated drug-disease associations in CTD (CTD mark is “M” or “M&T”).


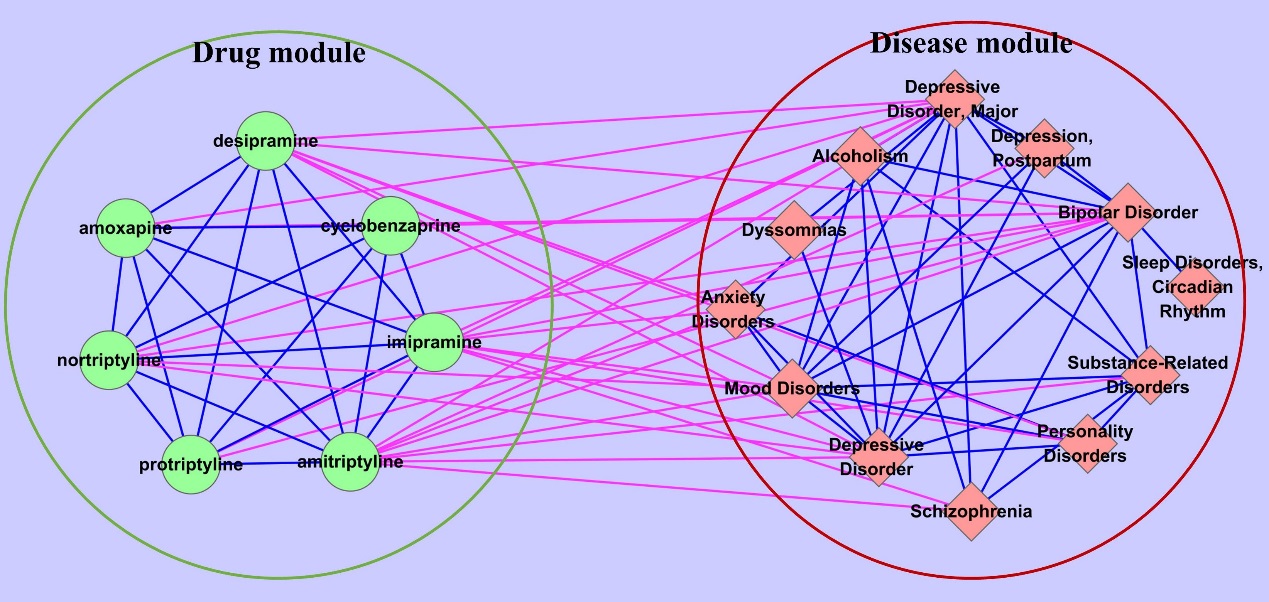


Supplementary Fig S2. The network topology of the third drug-disease module pair in Table 1 (Rank = 3). Internal connections within a module are labelled by blue, and external connections between two modules are labelled by purple. Green circle and red diamond nodes represent drugs and diseases respectively. The purple edges represent the reliably curated drug-disease associations in CTD (CTD mark is “M” or “M & T”).
